# Mindfulness-Based Intervention for the Reduction of Anxiety, Depression, and Stress Symptoms in Nursing Students: A Quasi-Experimental Study

**DOI:** 10.1192/j.eurpsy.2026.10749

**Published:** 2026-07-31

**Authors:** N. Aguayo-Verdugo, N. Ulloa-Chamblás, R. Zúñiga-Tapia, D. Barriga-Bustos, P. Bustamante-Barahona

**Affiliations:** 1 University of Glasgow, Glasgow, United Kingdom; 2 Universidad San Sebastian; 3 Universidad Andres Bello, Concepcion, Chile

## Abstract

**Introduction:**

University students, particularly in health-related fields, face rising levels of stress, anxiety, and depression, which negatively affect academic performance, social engagement, and overall well-being. Post-pandemic challenges, such as social isolation, uncertainty, and abrupt transitions to online learning, have exacerbated these issues. Mindfulness-based interventions (MBIs) have been reported to improve emotional regulation, resilience, and coping strategies in some university populations, although effects may vary depending on context, duration, and study design.

**Objectives:**

To evaluate the effectiveness of a mindfulness-based intervention in reducing stress, anxiety, and depression symptoms, among second-year nursing students at Universidad San Sebastián, Concepción, Chile.

**Methods:**

A quasi-experimental, non-randomised controlled study was conducted between August and December 2023. Out of 187 invited students, 155 participated; 66 were assigned to the intervention group and 89 to the control group. The intervention, delivered over 18 weeks, consisted of weekly 10–15 minute mindfulness sessions integrated into a course on communication and human interaction, the first nine weeks focused on guided breathing exercises, and the following nine weeks on body scan practices, delivered through instructional videos. The control group received the course as usual. Outcomes were measured using the DASS-21 scale. Data were analysed using a 2 (group: intervention vs. control) x 2 (time: pre vs. post) mixed ANOVA, with within-group effect sizes estimated using paired Cohen’s d.

**Results:**

At baseline, no statistically significant differences were observed between groups. The mixed ANOVA revealed no statistically significant group x time interactions for depression (p = .386), anxiety (p = .675), or stress (p = .167). However, within the intervention group, effect sizes indicated a small reduction in depression (d = –0.263), a moderate reduction in anxiety (d = –0.592), and a small-to-moderate reduction in stress (d = –0.339). These changes suggest potential benefits of mindfulness, despite not reaching statistical significance.

**Conclusions:**

The mindfulness-based intervention, embedded in nursing training, showed promising trends towards reducing stress and anxiety symptoms, with effect sizes pointing to clinically relevant improvements. While the findings did not achieve statistical significance, they highlight the potential value of incorporating mindfulness practices into undergraduate curricula. Further studies with larger samples and more rigorous designs are needed to strengthen the evidence base.

**Disclosure of Interest:**

None Declared

